# Supplementary material for: RNA surveillance via nonsense-mediated mRNA decay is crucial for longevity in daf-2/insulin/IGF-1 mutant C. elegans
Source: Nat Commun. 2017 Mar 9;8:14749. doi: 10.1038/ncomms14749 (PMC5347137; doi:10.1038/ncomms14749)
Supplement: Supplementary Information — Supplementary Figures, Supplementary Tables. [file ncomms14749-s1.pdf]

Supplementary Figure 1

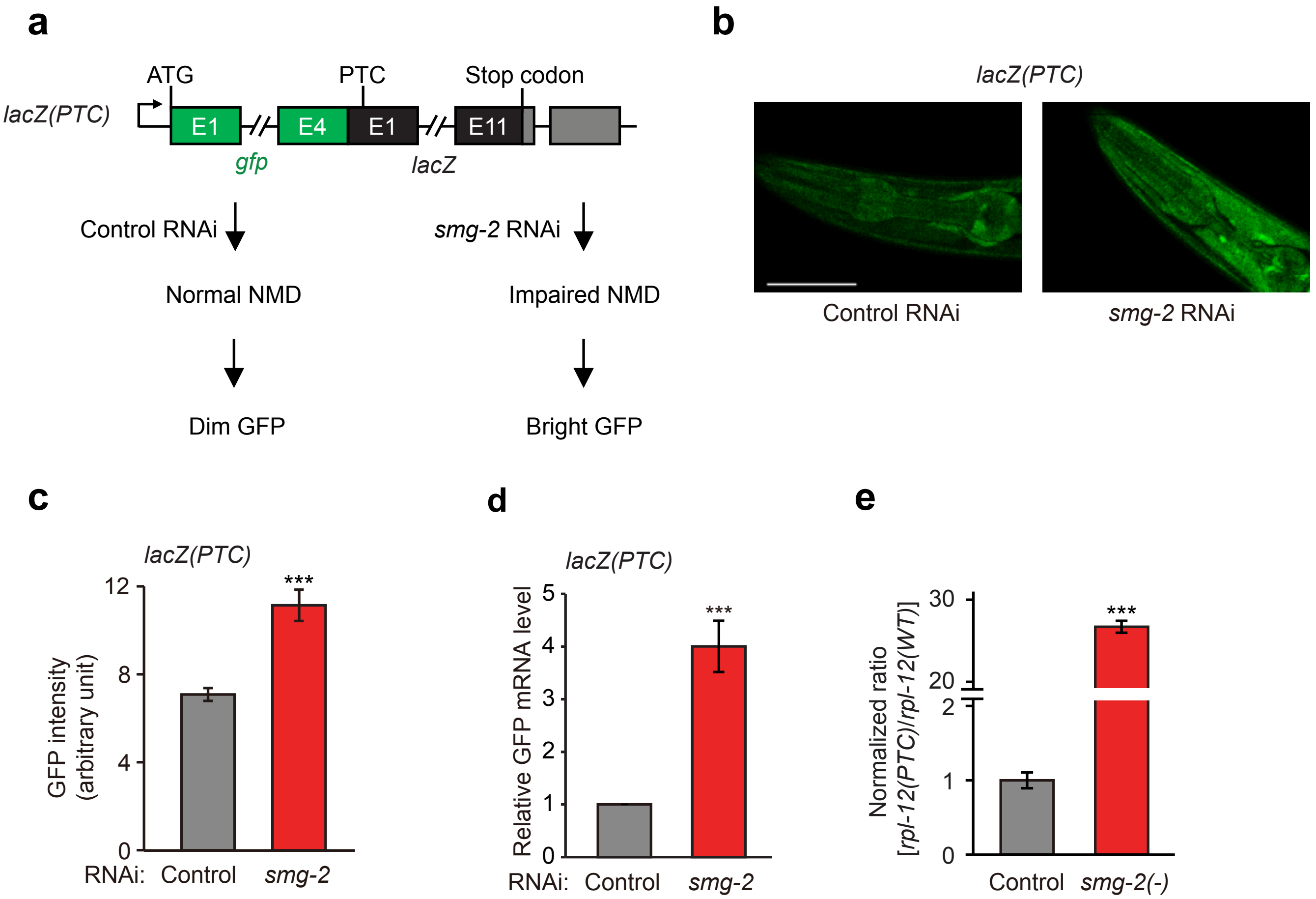

Supplementary Figure 1. PTC-containing NMD reporter GFP and *rpl-12* mRNA levels were increased by *smg-2* mutations. (**a**) A PTC-containing *gfp-lacZ* transgene, *lacZ(PTC)*[1], is an NMD reporter. The mRNA expressed from the transgene is degraded by NMD under normal conditions, and this results in dim GFP expression. When NMD is impaired, the GFP level is increased[1]. Green boxes represent *gfp* exons, black boxes represent *lacZ* exons, and gray boxes represent 3' UTRs. (**b**) Images of the NMD reporter, *sec-23p::gfp::lacZ(PTC)* (*lacZ(PTC)*), GFP-expressing *C. elegans* treated with control or *smg-2* RNAi (scale bar: 100 µm). (**c**) Quantification of data of *lacZ(PTC)*-expressing worms treated with control or *smg-2* RNAi (n ≥ 20 from two independent experiments). (**d**) qRT-PCR analysis for the *gfp* in the *lacZ(PTC)* animals treated with control or *smg-2* RNAi (n=2). (**e**) RT-PCR for PTC-containing *rpl-12* (*rpl-12(PTC)*) was normalized with *rpl-12* (*rpl-12(WT)*) transcripts, which do not contain PTC (n=3). Error bars represent SEM (two-tailed Student's t-test, ***$p < 0.001$).

Supplementary Figure 2

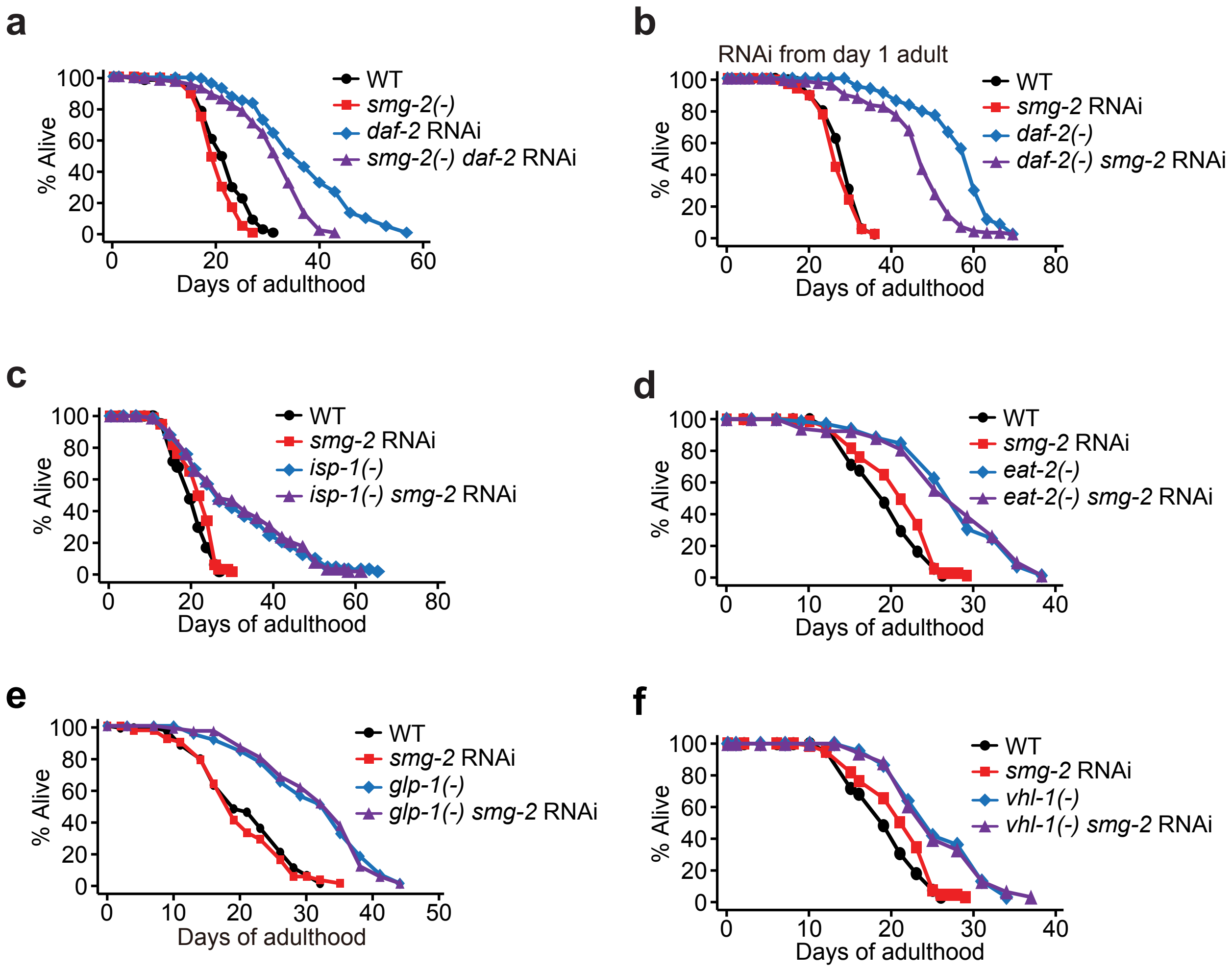

Supplementary Figure 2. Knockdown of *smg-2* had no effect on the long lifespan of the majority of longevity mutants except *daf-2* mutants. (**a**) *smg-2(qd101)* (*smg-2(-)*) mutations decreased the longevity induced by *daf-2* RNAi. (**b**) The lifespan of wild-type and *daf-2* mutant animals treated with *smg-2* RNAi from the first day of adulthood showed the partial requirement of *smg*-2 for the longevity of *daf-2(e1370)* (*daf-2(-)*) mutant animals. (**c-f**) The long lifespan induced by *isp-1(qm150)* (*isp-1(-)*) (**c**), *eat-2(ad1116)* (*eat-2(-)*) (**d**), *glp-1(e2141)* (*glp-1(-)*) (**e**), or *vhl-1(ok161)* (*vhl-1(-)*) (**f**) mutations was not changed by *smg-2* RNAi treatment. See Supplementary Data 1 for statistical analysis and additional repeats.

Supplementary Figure 3

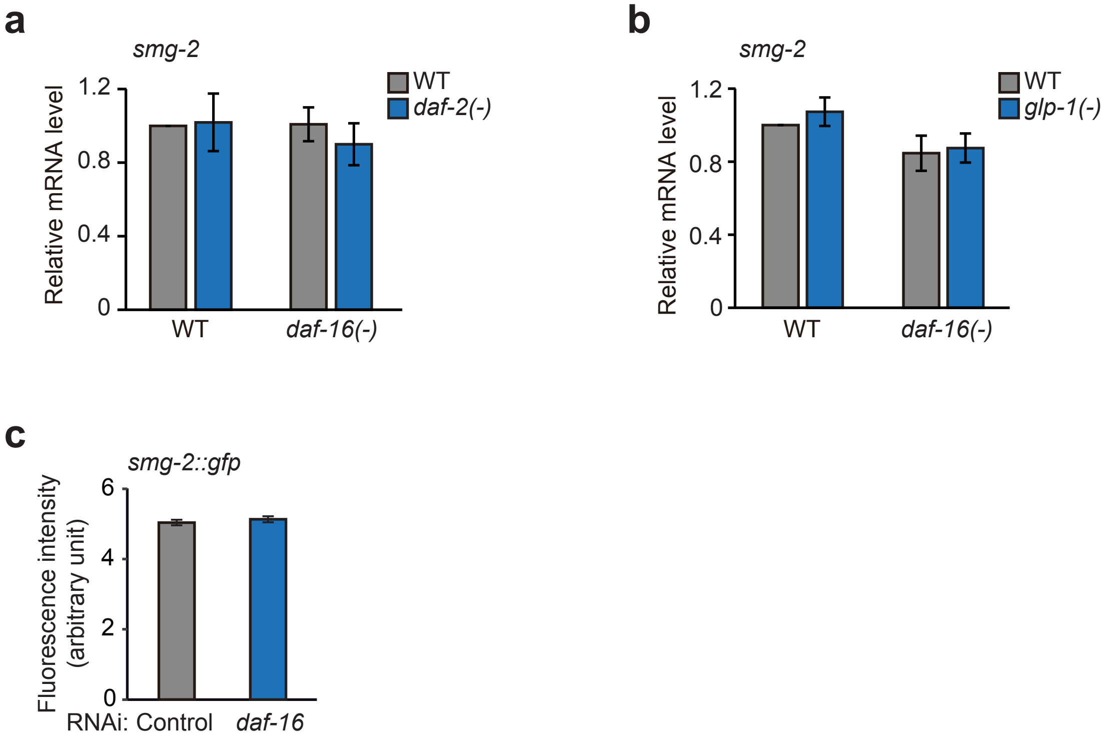

Supplementary Figure 3. The mRNA levels of *smg-2* were not changed by *daf-16, daf-2* or *glp-1* mutations. (**a, b**) qRT-PCR measuring the level of *smg-2* mRNA in wild-type (WT), *daf-2(e1370)*, *daf-16(mu86)*, or *daf-16(mu86); daf-2(e1370)* (n=4) (**a**), and in WT, *glp-1(e2141)*, *daf-16(mu86)*, or *daf-16(mu86); glp-1(e2141)* (n=4) (**b**) animals. (**c**) GFP intensities of *smg-2::gfp* animals treated with control or *daf-16* RNAi (n ≥ 43 from two independent experiments). Error bars represent SEM (two-tailed Student's t-test)

Supplementary Figure 4

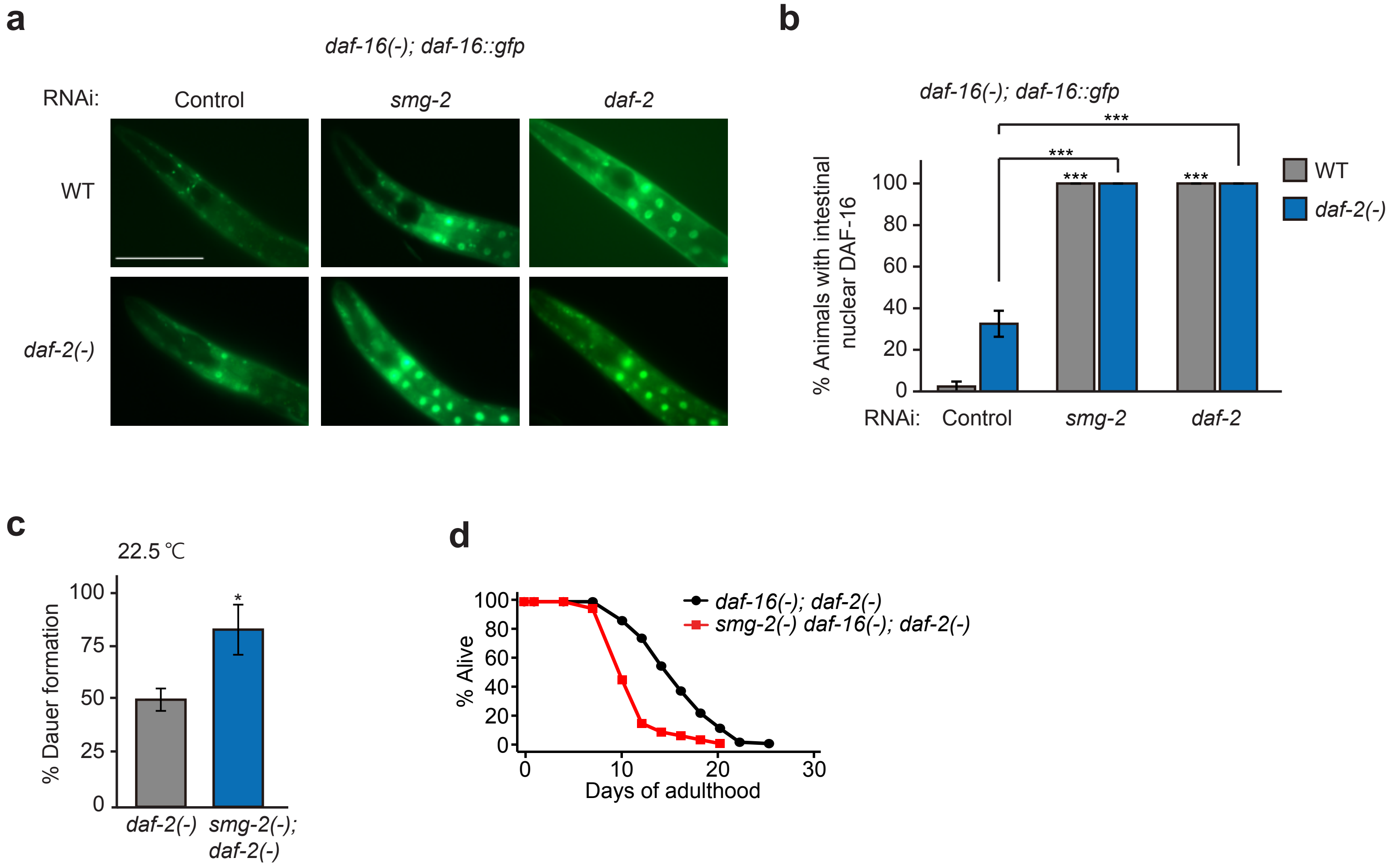

Supplementary Figure 4. DAF-16 activity appears to be increased by the genetic inhibition of *smg-2*. (**a**) Images of DAF-16::GFP in *daf-16(mu86)* (*daf-16(-)*) or *daf-16(mu86); daf-2(e1368)* (*daf-16(-); daf-2(-)*) mutant backgrounds upon knocking down *smg-2* (scale bar: 100 μm). *daf-2* RNAi was used as a positive control. (**b**) Quantification of the nuclear localized DAF-16::GFP in the intestinal cells in the panel **a** ($n \geq 37$ from three independent experiments). (**c**) Percentage of dauer at 22.5℃ of *daf-2(e1370)* and *smg-2(qd101); daf-2(e1370)* (*smg-2(-); daf-2(-)*) mutants. Error bars represent SEM (two-tailed Student's *t*-test, *$p < 0.05$, ***$p < 0.001$). (**d**) Mutations in *smg-2* decreased the lifespan of *daf-16(mu86); daf-2(e1370)* (*daf-16(-); daf-2(-)*) double mutants. See Supplementary Data 1 for statistical analysis and additional repeats.

Supplementary Figure 5

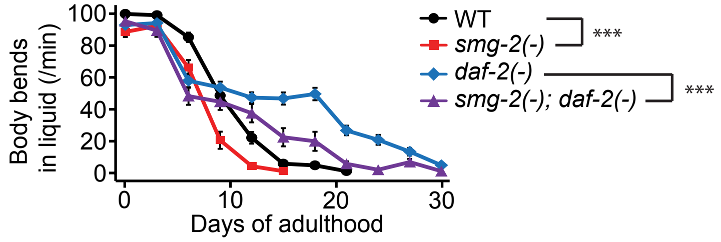

Supplementary Figure 5. Delayed age-dependent decreases in motility conferred by *daf-2* mutations were partially suppressed by *smg-2* mutations. Pooled data from two independent experiments of age-dependent declines in the motility of wild-type (WT), *smg-2(qd101)* (*smg-2(-)*), *daf-2(e1370)* (*daf-2(-)*) and *smg-2(qd101); daf-2(e1370)* (*smg-2(-); daf-2(-)*) animals. Error bars represent SEM. *p*-values were calculated by using two-way ANOVA test (***$p < 0.001$, n=30 from two independent experiments).

Supplementary Figure 6

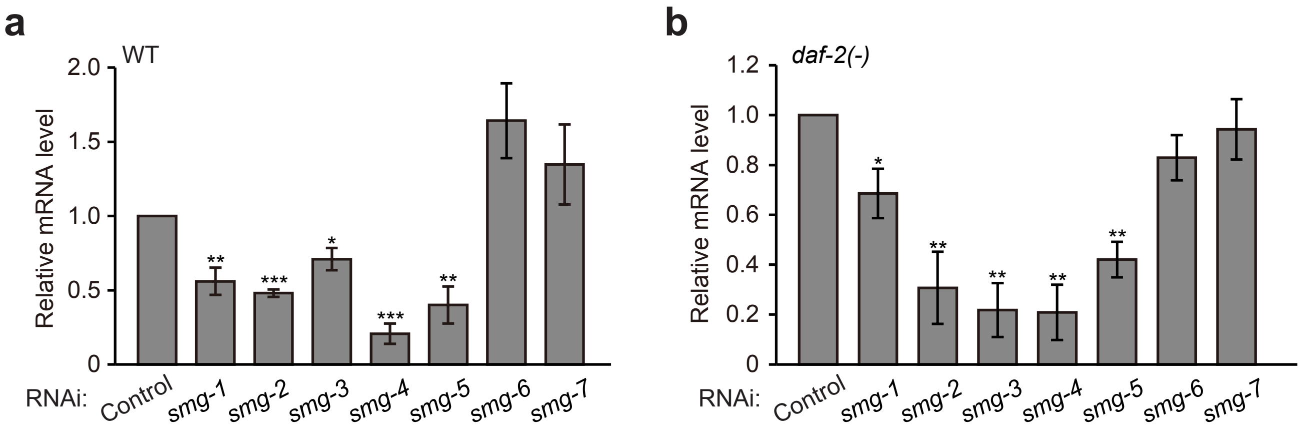

Supplementary Figure 6. The changes in mRNA levels of *smg* genes after each RNAi clone treatment. (**a,b**) The mRNA levels of *smg-1* through *smg-7* after treatment with each RNAi clone in wild-type (**a**) or *daf-2(e1370)* (*daf-2(-)*) mutants (**b**) were examined by using qRT-PCR ($n \geq 3$). Error bars represent SEM (two-tailed Student's t-test, *$p < 0.05$, **$p < 0.01$, ***$p < 0.001$).

Supplementary Figure 7

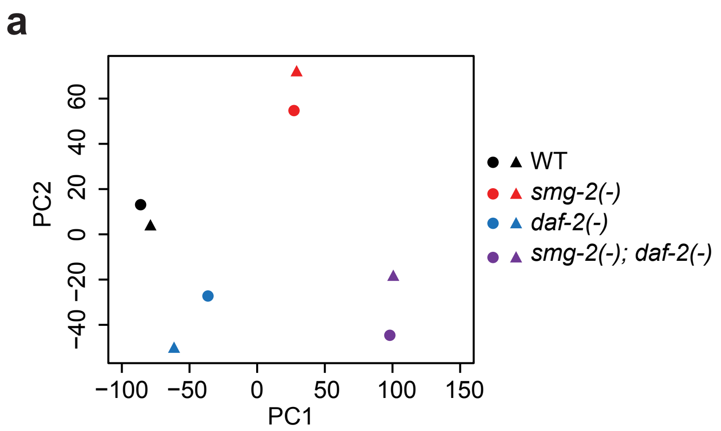

Supplementary Figure 7. Principal component analysis and analysis on overlap between genes that were induced by *smg-2* or *smg-1* mutations. (**a**) A principal component analysis (PCA) of mRNA sequencing data. Global gene expression patterns of biological duplicates for wild-type (WT), *smg-2(qd101)* (*smg-2(-)*), *daf-2(e1370)* (*daf-2(-)*), and *smg-2(qd101); daf-2(e1370)* (*smg-2(-); daf-2(-)*). Circles and triangles indicate the first and the second sets of biological replicates, respectively. PCA was performed for genes whose FPKM (fragments per kilobase of transcript per million) values were more than one. (**b**) Overlap between genes up-regulated by *smg-2(-)* mutations from our data and those by *smg-1(r861)* (*smg-1(-)*) mutations[2] was significant (RF: representation factor, hypergeometric probability test, ***$p < 0.001$).

Supplementary Figure 8

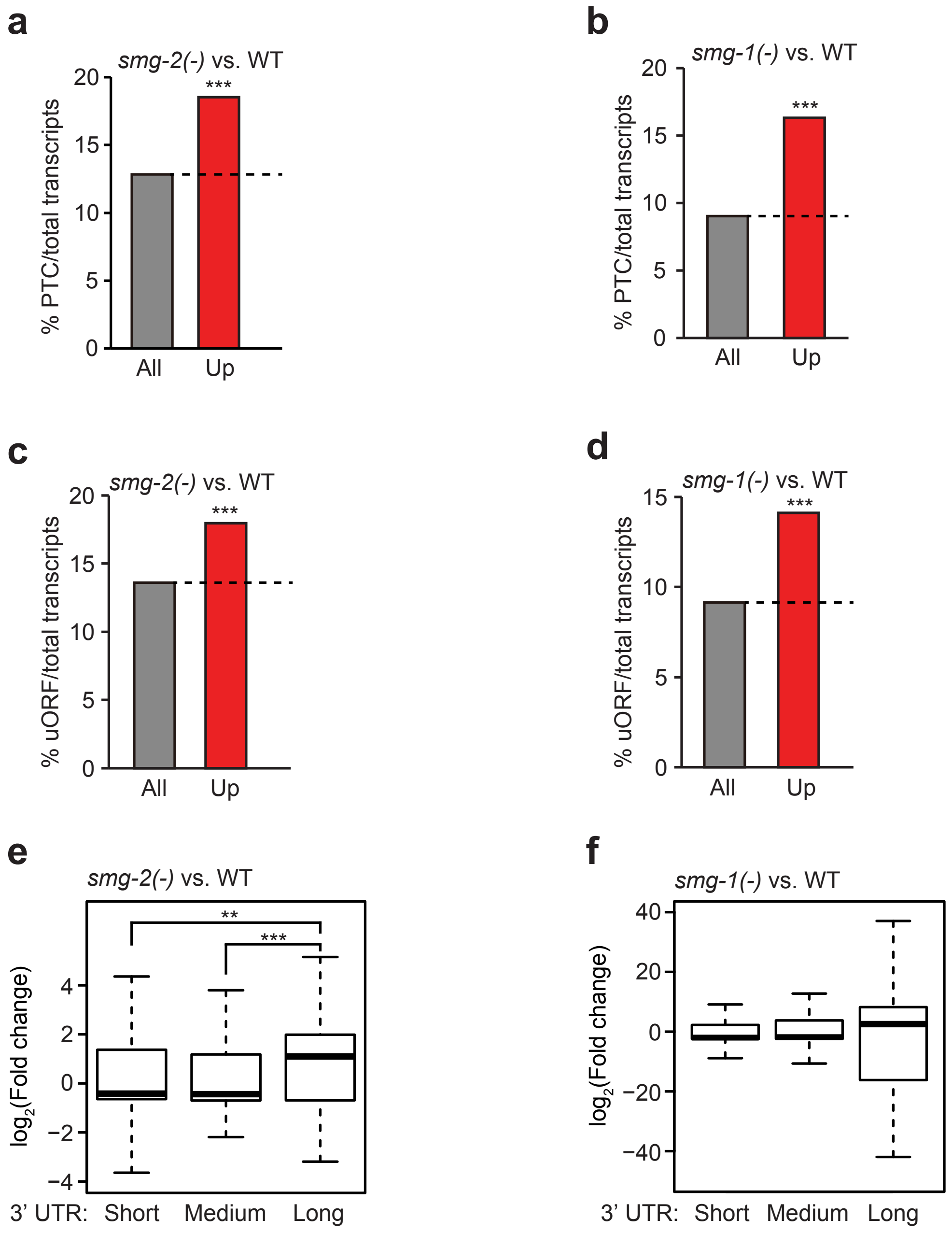

Supplementary Figure 8. Analysis of NMD targets in *smg-2* or *smg-1* mutants. (**a-d**) The fractions of PTC- (**a, b**) or uORF- (**c, d**) containing transcripts were increased among transcripts that were up-regulated (Up; $\log_2$ (fold change) ≥ 1, $p \leq 0.1$) by *smg-2(qd101)* (*smg-2(-)*) (**a**, **c**) or by *smg-1(r861)* ((*smg-1(-)*)[2] (**b**, **d**) mutations. Chi-square; ***$p < 0.001$. See Supplementary Table 1 and Data 3 for actual *p* values and the lists of PTC- or uORF-containing transcripts, respectively. (**e**, **f**) Box plots showing the fold changes of the transcripts that have different 3' UTR lengths between *smg-2(-)* and wild-type (**e**) or *smg-1(-)* and wild-type (**f**) animals. Short (≤ 350 nt), medium (350 nt < < 1500 nt), and long (≥ 1500 nt). 3' UTR lengths were defined as described previously[3]. Thick black lines indicate median values. Bottom and top of the box plot represent 25th and 75th percentile respectively, and whiskers indicate the data within the 1.5 interquartile range (IQR), which is the distance between the lower and upper quartiles of the data (**$p < 0.01$, ***$p < 0.001$, Wilcox rank sum test).

Supplementary Figure 9

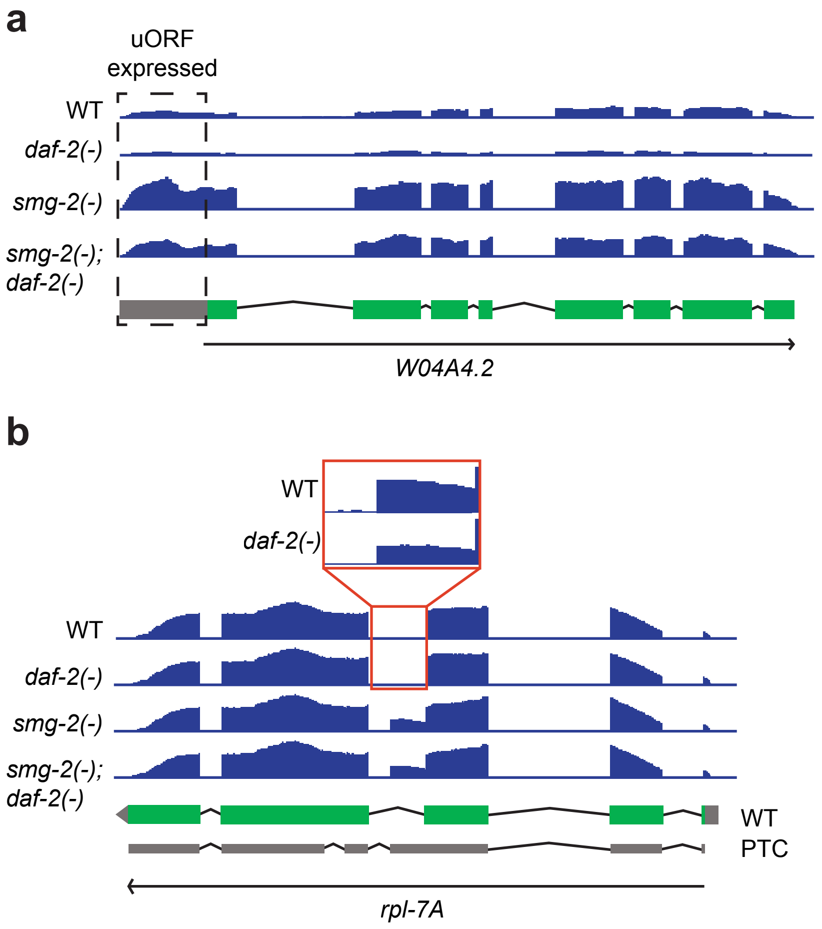

Supplementary Figure 9. Examples of NMD target transcript analysis. (**a**) The levels of uORF-expressing *W04A4.2* transcripts were decreased by *daf-2(e1370)* (*daf-2(-)*) mutations in a *smg-2*-dependent manner. (**b**) The transcript level of *rpl-7(PTC)*, a known endogenous NMD target[4], was decreased by *daf-2* mutations and increased by *smg-2(qd101)* (*smg-2(-)*) mutations. Green rectangles indicate exons, and grey parts indicate untranslated regions.

Supplementary Figure 10

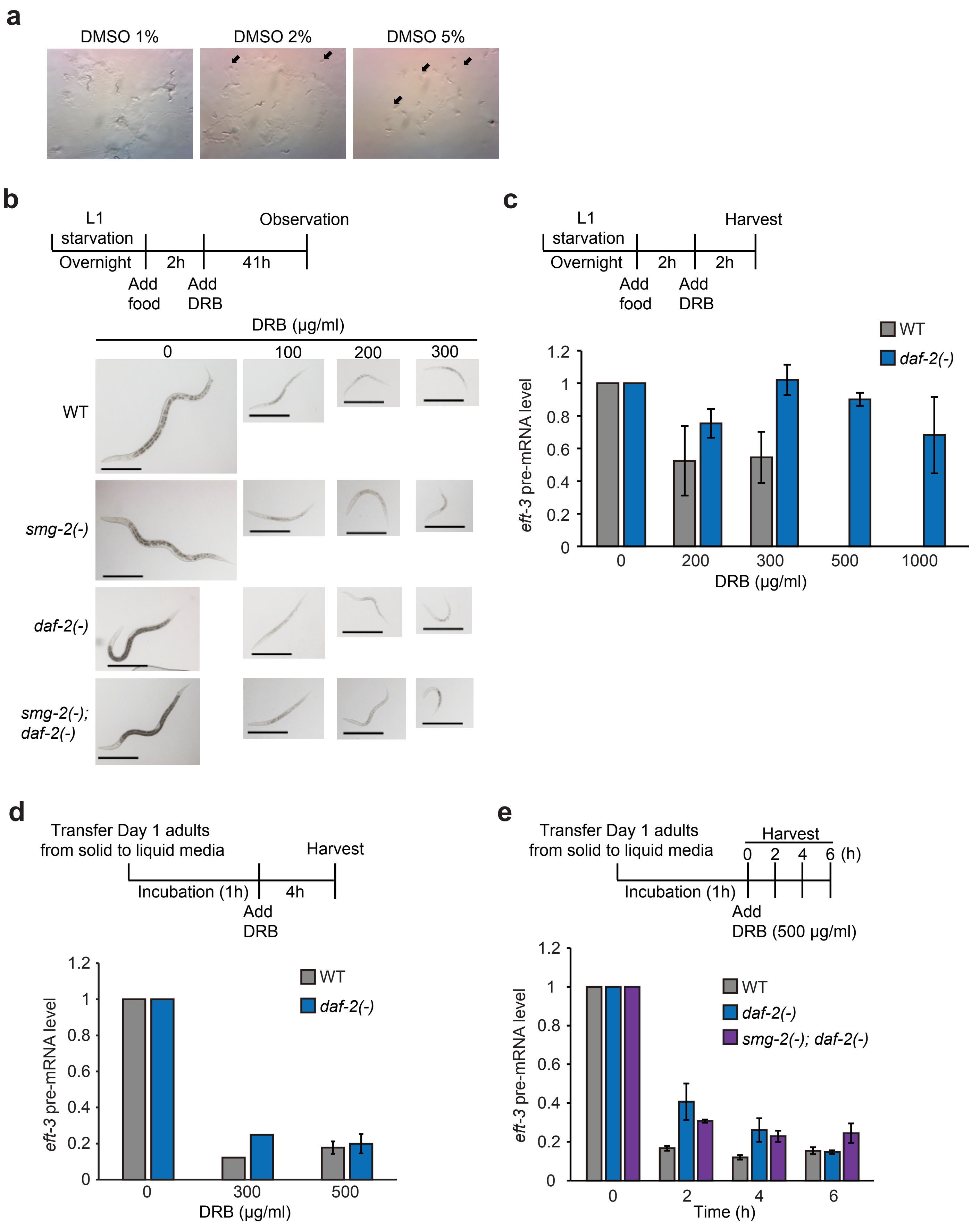

Supplementary Figure 10. Optimization of the experimental conditions for mRNA half-life assays using DRB. (**a**) Images of worms treated with various concentrations of DMSO. Arrows indicate developmentally arrested worms. (**b**) Images of wild-type (WT), *daf-2(e1370)* (*daf-2(-)*), *smg-2(qd101)* (*smg-2(-)*), and *smg-2(qd101); daf-2(e1370)* (*smg-2(-); daf-2(-)*) worms treated with DRB (5,6-dichlorobenzimidazole 1-β-D-ribofuranoside). Scale bar: 200 μm (**c**) The levels of *eft-3* pre-mRNA in L1 stage wild-type and *daf-2* mutant animals after treatment with DRB were determined by qRT-PCR (n=2). (**d**) The effects of DRB on *eft-3* pre-mRNA levels were examined in wild-type and *daf-2* mutant worms at day 1 adult stage by using qRT-PCR ($n \geq 1$). (**e**) The *eft-3* pre-mRNA levels after DRB treatment (500 μg/ml) at different time points (2, 4, 6 hours) were examined by using qRT-PCR (n=3).

Following are our descriptions regarding how we optimized experimental conditions for our RNA half-life assays. We treated worms with DRB, one of established transcription inhibitors used for measuring mRNA half-life[5, 6], to block transcription. As DRB is dissolved in DMSO, which can be toxic to worms[7], we first tested the effects of various concentrations of DMSO (1-5%) on the growth of worms. We found that 1% DMSO was the highest concentration that did not delay the development of *C. elegans* (Supplementary Fig. 10a). Next we tried to titrate optimal concentrations of DRB that inhibited growth, which reflects inhibited transcription[8], in wild-type, *daf-2*, *smg-2*, and *smg-2; daf-2* mutants. We found that over 200 μg/ml of DRB treatment was sufficient for inhibiting the growth (Supplementary Fig. 10b). Next, we measured the pre-mRNA levels of *eft-3*, (a positive control) by using qRT-PCR to determine whether treatments with DRB inhibited transcription as previously described[8]. We found that 200 μg/ml of DRB partially decreased transcription in wild-type, but did not in *daf-2* mutants (Supplementary Fig. 10c). A much higher concentration of DRB (1000 μg/ml of DRB) inhibited

transcription in *daf-2* mutants, whereas the same concentration of DRB killed wild-type worms. Thus, we concluded that L1 larval worms were not suitable for our mRNA half-life assays that require comparing *daf-2* mutants and wild-type animals. We therefore changed our strategy to examine the RNA half-lives of adult worms. We found that 500 μg/ml of DRB treatment successfully inhibited transcription in wild-type, *daf-2(-)*, and *smg-2(-); daf-2(-)* mutant animals, although it seems that *daf-2* mutants are more resistant to DRB than wild-type at early time points (2 hr and 4 hr) (Supplementary Fig. 10d,e).

Supplementary Figure 11

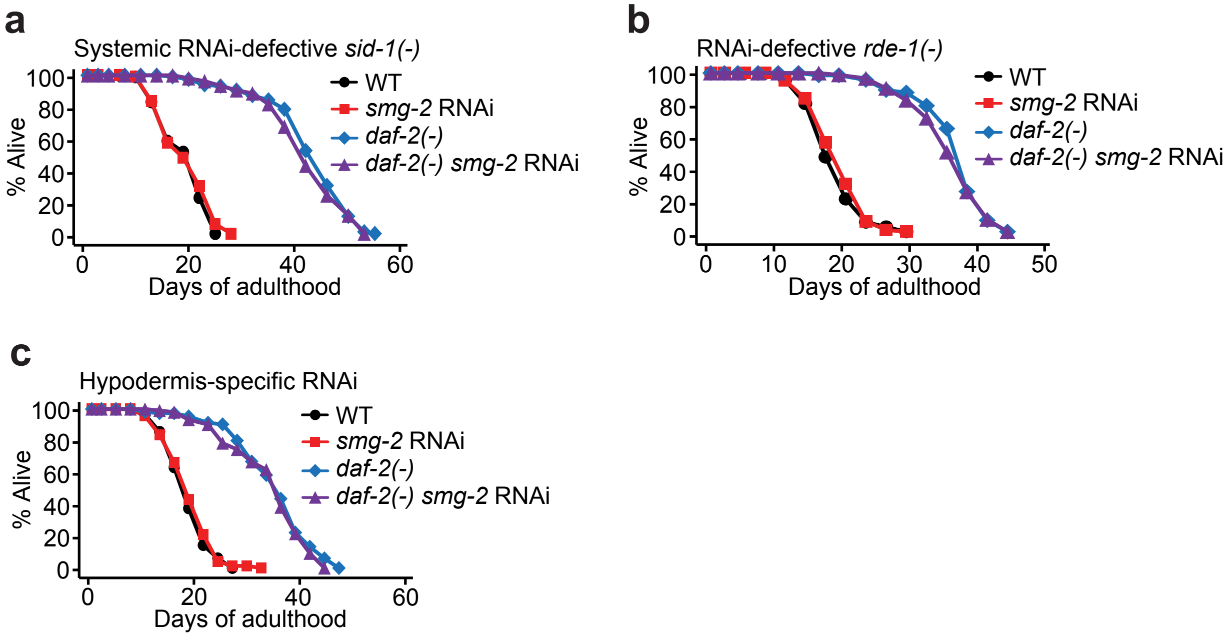

Supplementary Figure 11. Double-stranded RNA of *smg-2* did not affect the lifespan of RNAi-defective or hypodermis-specific RNAi strains. (**a**) *smg-2* double-stranded RNA (dsRNA) treatment did not affect lifespan in systemic-RNAi defective *sid-1(pk3321)* (*sid-1(-)*) or *daf-2(e1370); sid-1(pk3321)* (*daf-2(-)*) mutants. (**b**) The lifespan of RNAi-defective *rde-1(ne219)* (*rde-1(-)*) or *daf-2(e1370); rde-1(ne219)* (*daf-2(-)*) animals was not influenced by *smg-2* dsRNA treatment. (**c**) A hypodermis-specific *smg-2* RNAi had no effect on the lifespan of *daf-2(e1370); rde-1(ne219); Is[wrt-2p::rde-1; myo-2p::rfp]* (*daf-2(-)*) mutants. See Supplementary Data 1 for statistical analysis and additional repeats.

Supplementary Figure 12

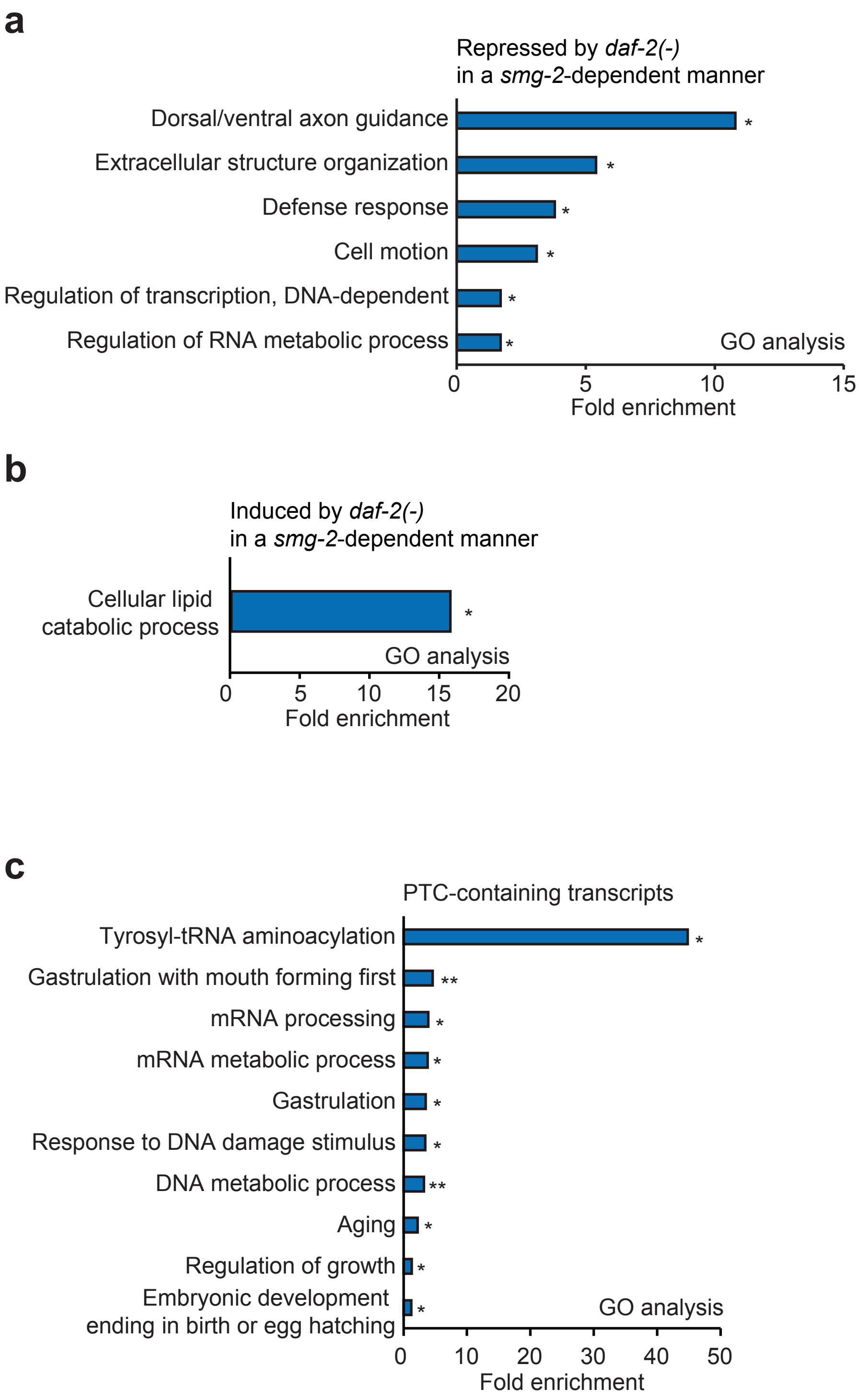

Supplementary Figure 12. Gene ontology analysis of NMD target transcripts (**a, b**) Gene ontology (GO) analysis of genes that were down- ($\log_2$ (fold change) ≤ -0.4) (**a**), or up-regulated (**b**) ($\log_2$ (fold change) ≥ 0.4, $*p < 0.05$, $**p < 0.01$ modified Fisher's exact tests[9]) in *daf-2(e1370)* (*daf-2(-)*) mutants compared to those in wild-type in a *smg-2*-dependent manner. (**c**) GO terms that are enriched among PTC-containing transcripts whose levels were decreased by *daf-2* mutations in a *smg-2*-dependent manner ($\log_2$ (fold change) ≤ -0.4, $*p < 0.05$, $**p < 0.01$, modified Fisher's exact tests[9]). See Supplementary Data 2 for the lists of genes or transcripts that were used for GO analysis.

Supplementary Figure 13

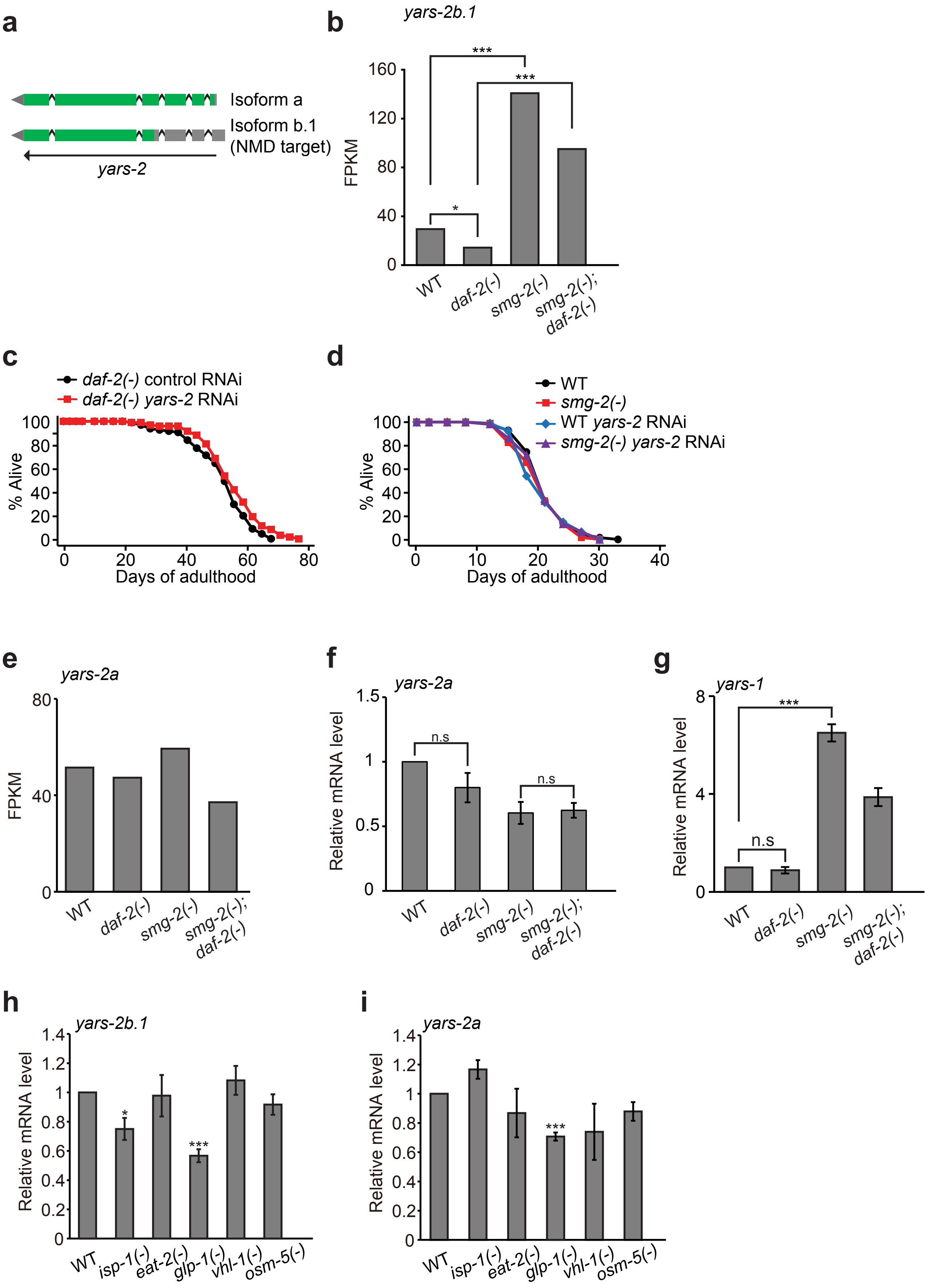

Supplementary Figure 13. Characterization of tRNA synthetase *yars-2* as a crucial NMD target. (**a**) A diagram for *yars-2* isoforms. Among four isoforms of *yars-2,* two isoforms (*yars-2a* and *yars-2b.1*) that were found to be sufficiently detected (FPKM > 20 in wild-type worms) in our data were presented. Green rectangles indicate exons, and grey parts indicate untranslated regions. (**b**) The mRNA levels of *yars-2b.1* in wild-type (WT), *daf-2(e1370)* (*daf-2(-)*), *smg-2(qd101)* (*smg-2(-)*), and *smg-2(qd101); daf-2(e1370)* (*smg-2(-); daf-2(-)*) obtained by using mRNA sequencing (cuffdiff, *$p < 0.05$, ***$p < 0.001$). FPKM stands for fragments per kilobase of exon per million fragments mapped. (**c,d**) The effects of *yars-2* RNAi on the lifespan of *daf-2(-)* (**c**), wild-type, or *smg-2(-)* (**d**) mutants. *yars-2* RNAi has been shown to increase lifespan in *rrf-3(pk1426)* mutant backgrounds[10], and we obtained consistent results (data not shown). See Supplementary Data 1 for statistical analysis and additional repeats. (**e,f**) RNA seq. (**e**) or qRT-PCR ($n \geq 3$) (**f**) results for the mRNA levels of *yars-2a* isoform, which is not a target of NMD. (**g**) The mRNA levels of *yars-1* were examined by qRT-PCR ($n \geq 3$). (**h,i**) The mRNA levels of *yars-2b.1* (**h**) or *yars-2a* (**i**) in *isp-1(qm150)* (*isp-1(-)*), *eat-2(ad1116)* (*eat-2(-)*), *glp-1(e2141)* (*glp-1(-)*), *vhl-1(ok161)* (*vhl-1(-)*) or *osm-5(p813)* (*osm-5(-)*) were examined by qRT-PCR ($n \geq 4$). Error bars represent SEM (two-tailed Student's t-test, *$p < 0.05$, ***$p < 0.001$).

Supplementary Tables

Supplementary Table 1. ***p*** **values for analysis of PTC- or uORF-containing transcripts related to Fig. 4, 6, and Supplementary Fig. 8**

p values for transcripts containing PTCs or uORFs			PTCs	Figure in text	uORFs	Figure in text
Condition	*daf-2(-)* vs. WT	Down	0.0425	Fig. 4a	0.0037	Fig. 4b
	Neuronal *daf-2(-)* vs. WT	Down	0.0033	Fig. 6i	0.0160	Fig. 6j
	smg-2(-) vs. WT	Up	<0.0001	Supplementary Fig. 8a	<0.0001	Supplementary Fig. 8c
	smg-1(-) vs. WT	Up	<0.0001	Supplementary Fig. 8b	<0.0001	Supplementary Fig. 8d

p values were calculated against 'All' conditions using Chi-square test with Yate's correction by Graphpad (an online resource for statistical test; http://graphpad.com/quickcalcs/contingency1.cfm).

Supplementary Table 2. The list of oligonucleotides used for the quantitative RT-PCR

ama-1-F-TGGAACTCTGGAGTCACACC

ama-1-R-CATCCTCCTTCATTGAACGG

gfp-F-GCACAAATTTTCTGTCAGTGG

gfp-R-GACAAGTGTTGGCCATGGAAC

pmp-3-F-GTTCCCGTGTTCATCACTCAT

pmp-3-R-ACACCGTCGAGAAGCTGTAGA

rpl-12(PTC)-F-GTGATTTTCATGCTCCTGAAG

rpl-12(PTC)-R-CAAGGATCTCCTTGACGG

rpl-7A(PTC)-F-GACAGCCAGTCCGGTTGG

rpl-7A(PTC)-R-GTCTAGTTCACCTATCAGAGTAAATG

smg-1-F-GGGAACCTGATAGAACAGTTTC

smg-1-R-CTCTACATTTTTCGTAGTTTGAAG

smg-2-F-GATTGCTGAGAGCCCGGAGAG

smg-2-R-GAACAGATAGCCAATGTGCTCC

smg-3-F-CAATTGAAACTATTCGAATATC

smg-3-R-CTGTAGAGCTCTGCAAGATAAAC

smg-4-F-CTGTTGGCGAGCAACAAGGC

smg-4-R-TCAGTTTGGTGGTGA

smg-5-F-CGATGTTGCGCAGAAAAGGC

smg-5-R-GATTGAACTTGTACAGTCC

smg-6-F-GTGATGTGATGATCTACCGCCC

smg-6-R-CCAAATATTGAACAAGAAATGCGTGGG

smg-7-F-CAGTCGAAACGGATGAAGATG

smg-7-R-GCATGAAAAGAAAAAAAACCAAAAATTATGAC

yars-1-F-GGTCAATTTACTGGAAAAAGCTAC

yars-1-R-GGAGCTCAGCGATGG

yars-2a-F-CGTGCACCTCGAGAAAG

yars-2a-R-CAGCAGGTCCGTTGG (same as *yars-2b.1*-R)

yars-2b.1-F-GTGGCCTTAAAAACGAGAAAG

yars-2b.1-R-CAGCAGGTCCGTTGG (same as *yars-2a*-R)

18S rRNA-F-CAGACCAAACGTTTTCGGACGTTG

18S rRNA-R-TTGGACGTGGTAGCCGTTTCTAAG

Supplementary Table 3. The list of oligonucleotides used for the RT-PCR

rpl-12 RT-F-ACCCAAGACTGGAAGGGTCT

rpl-12 RT-R-GCCATCGATCTTGGTCTCAT

Supplementary References

1. Longman, D., Plasterk, R. H., Johnstone, I. L., Caceres, J. F. Mechanistic insights and identification of two novel factors in the *C. elegans* NMD pathway. *Genes & development* **21**, 1075-1085 (2007).

2. Ramani, A. K. *et al.* High resolution transcriptome maps for wild-type and nonsense-mediated decay-defective *Caenorhabditis elegans*. *Genome biology* **10**, R101 (2009).

3. Fanourgakis, G. & Lesche, M. Chromatoid Body Protein TDRD6 Supports Long 3' UTR Triggered Nonsense Mediated mRNA Decay. **12**, e1005857 (2016).

4. Mitrovich, Q. M. & Anderson, P. Unproductively spliced ribosomal protein mRNAs are natural targets of mRNA surveillance in C. elegans. *Genes & development* **14**, 2173-2184 (2000).

5. Bensaude, O. Inhibiting eukaryotic transcription: Which compound to choose? How to evaluate its activity? *Transcription* **2**, 103-108 (2011).

6. Chen, C. Y., Ezzeddine, N., Shyu, A. B. Messenger RNA half-life measurements in

mammalian cells. *Methods in enzymology* **448**, 335-357 (2008).

7. Solis, G. M. & Petrascheck, M. Measuring *Caenorhabditis elegans* life span in 96 well microtiter plates. *Journal of visualized experiments : JoVE*, doi: 10.3791/2496 (2011).

8. Miki, T. S., Ruegger, S., Gaidatzis, D., Stadler, M. B., Grosshans H. Engineering of a conditional allele reveals multiple roles of XRN2 in *Caenorhabditis elegans* development and substrate specificity in microRNA turnover. *Nucleic acids research* **42**, 4056-4067 (2014).

9. Huang da, W., Sherman, B. T., Lempicki, R. A. Systematic and integrative analysis of large gene lists using DAVID bioinformatics resources. *Nature protocols* **4**, 44-57 (2009).

10. Kim, Y. & Sun, H. Functional genomic approach to identify novel genes involved in the regulation of oxidative stress resistance and animal lifespan. *Aging cell* **6**, 489-503 (2007).
